# A Case of Breast Cancer with Cystic Axillary Lymph Node Metastasis

**DOI:** 10.70352/scrj.cr.25-0163

**Published:** 2025-10-31

**Authors:** Mariko Yoshino, Yoshiya Horimoto, Mutsumi Hayashi, Yuko Ueki, Yumiko Ishizuka, Hiroko Onagi, Takuo Hayashi, Kotaro Iijima, Goro Kutomi

**Affiliations:** 1 Department of Breast Oncology, Faculty of Medicine, Juntendo University, Tokyo, Japan; 2 Department of Breast Surgery and Oncology, Tokyo Medical University, Tokyo, Japan; 3 Department of Human Pathology, Faculty of Medicine, Juntendo University, Tokyo, Japan

**Keywords:** encapsulated papillary carcinoma, breast cancer, axillary lymph node metastasis, cystic mass

## Abstract

**INTRODUCTION:**

Encapsulated papillary carcinoma (EPC) is a relatively rare form of breast cancer and is often low grade. Even in cases of EPC with invasion, it rarely metastasizes. We herein report a case of EPC with invasion that presented with a cystic axillary lymph node metastasis that was highly characteristic on imaging.

**CASE PRESENTATION:**

A 73-year-old woman presented with a mass in the right breast. Mammogram showed linear calcifications in the middle-outer region of the right breast and a lobulated mass in the lower region. Ultrasound revealed a cystic mass with extensive fluid formation in the breast and a hypoechoic mass with indistinct borders. Numerous cystic enlarged lymph nodes, similar in appearance to the intramammary mass, were detected in the ipsilateral axilla. Contrast-enhanced MRI also revealed multiple cystic enlarged lymph nodes in the axilla. Cytology of the axillary lymph nodes was suggestive of metastasis. The patient underwent a right mastectomy and axillary dissection, and histopathological examination confirmed a diagnosis of EPC with invasion (pT2N2aM0, Stage IIIA). The metastatic axillary lymph nodes contained cystic structures with papillary proliferation, closely resembling the primary EPC lesion. The patient was treated postoperatively with endocrine therapy alone, and to date, no recurrence has been observed 18 months after surgery.

**CONCLUSIONS:**

This case highlights a rare instance of EPC with invasion presenting as cystic lymph node metastases, which was strikingly evident on imaging. Awareness of this unique metastatic pattern may be useful in daily clinical practice for facilitating accurate diagnosis and appropriate management of EPC.

## Abbreviations

EPC
: encapsulated papillary carcinoma
ER
: estrogen receptor
HER2
: human epidermal growth factor receptor 2
PgR
: progesterone receptor

## INTRODUCTION

Encapsulated papillary carcinoma (EPC) is relatively rare, accounting for 0.5%–2% of breast cancers.^1)^ It occurs most often in postmenopausal women, is hormone receptor positive, HER2 negative, and low grade in the majority of cases,^2)^ and is classified as a noninvasive cancer despite the absence of peritumoral myoepithelium.^3)^ Although it is treated like invasive carcinoma when accompanied by surrounding frank invasion, the prognosis is good, and lymph node and distant metastases are unlikely to occur.^4)^ On imaging, it often shows a relatively well-defined, round or oval solid cystic appearance.^5)^

In the present report, we describe a case of EPC with invasion that presented with a cystic axillary lymph node metastasis that was very characteristic on imaging.

## CASE PRESENTATION

A 73-year-old woman presented to our hospital with a complaint of a mass in the right breast. Nine years earlier, she had undergone incisional drainage of a subcutaneous abscess in the lower outer quadrant of the right breast, and she had been aware of a mass in the same area for two years, which had been growing. On physical examination, a well-defined mass of 35 mm in the lower inner quadrant of the right breast and 30 mm under the incision scar in the lower outer quadrant were palpable.

**Fig. 1:**
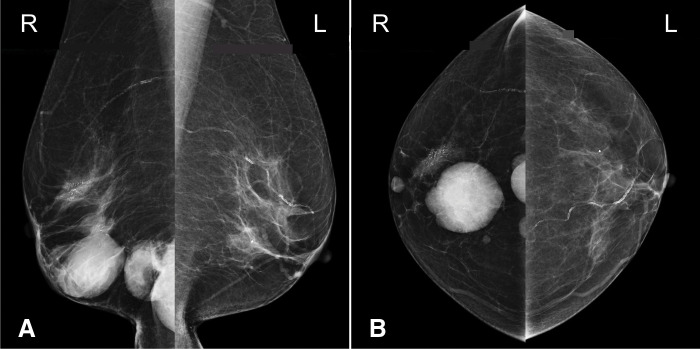
Mammogram findings. Linear calcification was observed in the middle part of the right breast in the MLO view (**A**) and in the outer part in the CC view (**B**). Both findings were classified as Category 5. Well-defined masses were also observed in the lower part. There was no specific finding in the left breast (Category 1). CC, craniocaudal; MLO, mediolateral oblique

**Fig. 2:**
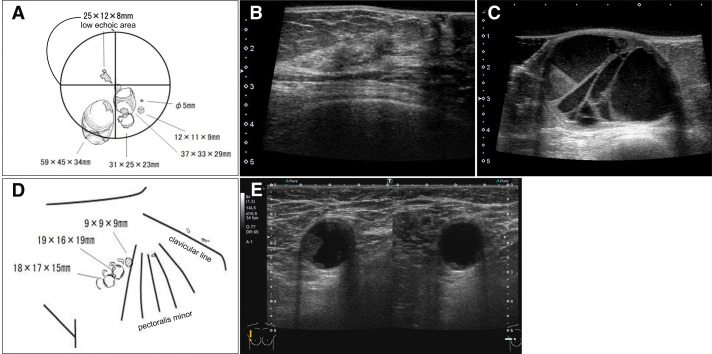
Ultrasound image and schema at initial presentation. (**A**) Schematic diagram of the right breast findings. (**B**) A hypoechoic area as large as 25 mm is seen in the upper outer quadrant of the right breast, and (**C**) a cystic mass with fluid formation is extensively involved in the central and lower parts of the right breast. (**D**) Schematic diagram of the right regional lymph node findings. (**E**) A large cystic lymph node is seen in the axilla as well as in the breast.

**Fig. 3:**
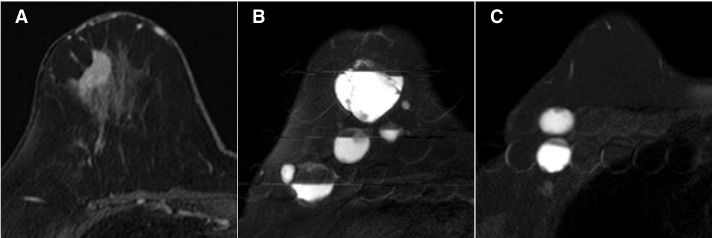
Contrast-enhanced MRI of the breast. (**A**) A 27 × 15-mm early darkened mass lesion extending toward the nipple in the lateral superior region of the right breast was observed, which was considered to be consistent with the calcified area on the mammogram and the hypoechoic area noted on the ultrasound. (**B**) Numerous cystic masses are present in the nipple to the lateral inferior region, with an internal T2 high-signal fluid formation. (**C**) Similar cystic masses are present in the axilla.

Initial mammogram showed a zoned linear calcification in the middle-outer region of the right breast and a lobulated mass in the lower region (**Fig. 1**). Ultrasound revealed an extensive cystic mass with fluid formation in a large area covering the central and lower parts of the right breast and an indistinct hypoechoic mass 25 mm in size in the upper outer quadrant of the right breast. In the ipsilateral axilla, there were numerous cystic enlarged lymph nodes similar to the intramammary mass (**Fig. 2**). Contrast-enhanced MRI of the breast showed a 27 × 15-mm early darkened mass lesion extending toward the nipple in the lateral superior portion of the right breast, consistent with the calcified area on the mammogram (**Fig. 3**). Numerous cystic masses were present in the nipple to the lateral inferior region, with internal fluid formation. Multiple masses in the axilla were similarly cystic in appearance.

A needle biopsy was performed on a cystic mass in the lower outer quadrant of the right breast, but no obvious malignant findings were observed, only fibrous tissue. A second needle biopsy was performed on the mass in the upper outer quadrant of the right breast, and the diagnosis of invasive ductal carcinoma of the breast (ER-positive, PgR-positive, HER2: 1+) was made. Cytology performed on the axillary lymph node showed a small number of cells, but a mass of atypical epithelial cells was found, which was suspicious for malignancy. Based on the above examination results, a diagnosis of right invasive ductal carcinoma of the breast, cT2N1M0, Stage IIB, was made, and right mastectomy and axillary lymph node dissection (Level I) were performed.

**Fig. 4:**
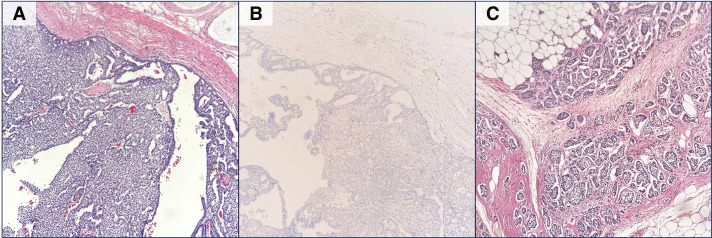
Histological findings of the primary breast lesion (surgical specimen). (**A**) EPC component of the primary breast lesion (hematoxylin and eosin staining). Tumor cells proliferate in a papillary pattern within cystic structures. (**B**) Immunohistochemistry of p63, a marker for myoepithelial cells, in the same region shows that myoepithelial cells are absent at the tumor periphery. (**C**) Frank invasion into the stroma and adipose tissue is observed outside the cystic structures. EPC, encapsulated papillary carcinoma

**Fig. 5:**
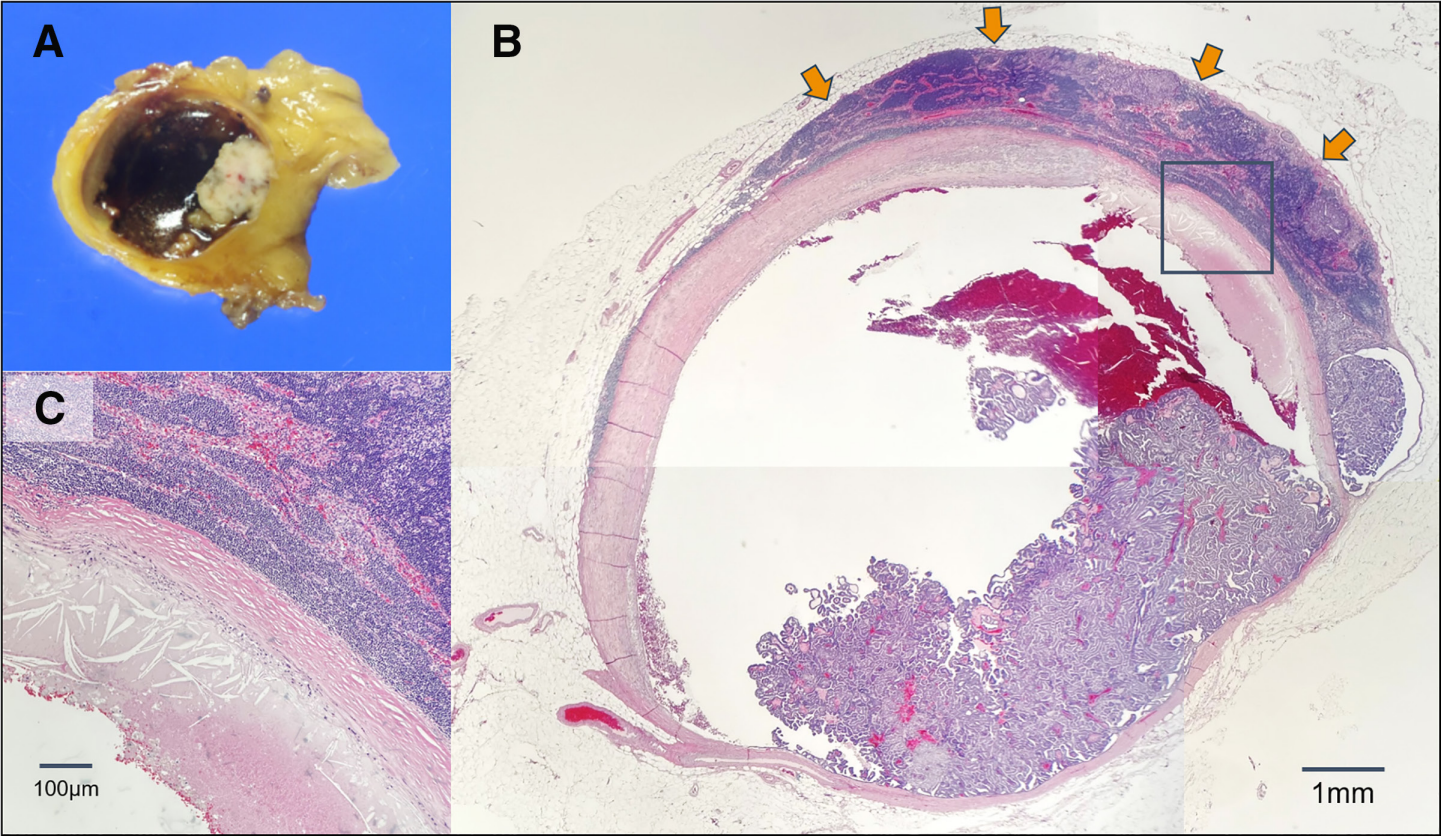
Histological findings of lymph node metastasis (surgical specimen). (**A**) Cut surface of the lymph node metastasis. (**B**) Low-power view of the lymph node metastasis (hematoxylin and eosin staining). The arrows indicate the residual normal lymph node structures surrounding the metastatic lesion. (**C**) Fibrous capsule-like structure at the margin of the metastatic lesion.

Postoperative histological findings showed papillary growth of atypical cells in a cyst-like structure with internal hemorrhage in an area of 70 × 60 × 20 mm, which was consistent with EPC (**Fig. 4**). The lesion was surrounded by invasive ductal carcinoma of the breast over an area of 30 × 17 × 10 mm. The axillary lymph nodes showed numerous metastatic findings (5 of 19). **Figure 5** shows the gross and histological images. Hemorrhage and papillary growth of tumor components were observed inside the cystic structure, which resembled the histology of the primary EPC. The final pathological diagnosis was EPC with invasion, NG1, Ki-67 labeling index 20%, ER 100%, PgR 80%, HER2:2+ (fluorescence *in situ* hybridization negative), pT2N2aM0, Stage IIIA.

Postoperative treatment consisted of endocrine therapy (anastrozole) only, taking into account the age and grade of the tumor, and no apparent recurrence has been observed one and a half years after the surgery.

## DISCUSSION

The frequency of lymph node metastasis in EPC is low, ranging from 0% to 6% even when accompanied by invasive cancer.^2,6,7)^ Metastasis may be suspected when atypical lymph node imaging findings, such as in this case, are observed in clinical practice. However, if the primary tumor has the histology of an EPC, the metastatic lesion is likely to retain a similar structural pattern. Indeed, it has been reported that lymph node metastases of EPC exhibit a papillary architecture.^3)^ Therefore, it is essential to properly understand the histology of the needle biopsy. Furthermore, EPCs may form similar structures in distant metastases. Kitahara et al. reported a case of distant metastatic EPC,^5)^ but did not discuss the imaging findings of the metastases. Pareja et al. conducted a paired analysis of primary and metastatic lesions in special subtypes of breast cancer and demonstrated that both share highly similar somatic mutation profiles.^8)^ Such molecular-level similarity supports the notion that the differentiation tendency and structural characteristics of the primary tumor are preserved in the metastatic sites. Cyst formation presumably results from factors such as the secretory activity of tumor cells and the formation of cystic spaces due to papillary architecture. In the present case, we suspect that the cystic changes observed in the metastatic lesion reflect the structural properties of the primary tumor.

When cytology was performed preoperatively on the axillary lymph node in this case, the internal component of the cyst was punctured with great care in order to obtain the correct diagnostic quality. If the cytological diagnosis had been inconclusive because no cellular component was obtained and only fluid from the cystic component was drawn, additional work would have been required, such as preoperative needle biopsy of the lymph node or intraoperative lymph node sampling, for a rapid diagnosis. The same event would have occurred if the EPC had metastasized to distant sites and showed cyst-like metastases on imaging. A needle biopsy may not collect enough cells to make an accurate diagnosis, and a surgical biopsy may need to be considered.

This case is very interesting considering the growth pattern of EPC. In the metastatic lymph node nests, cystic-like structures with fibrous walls, which are not present in normal lymph nodes, were formed. This structure is thought to be newly formed with the proliferation of EPCs and has been described in several previous reports.^6,9,10)^ In one of these reports, basement membrane-like structures were found to be positive for type IV collagen and laminin.^6)^ This phenomenon may also apply to EPCs in primary lesions. In other words, the fibrous capsule-like structures surrounding EPCs may not be the original cyst wall, but may have been newly formed by interaction with the surrounding stroma during the process of compressible growth of the EPCs.^6)^

Postoperative adjuvant therapy for EPC with invasion should follow the same principles as those for conventional invasive breast cancer. According to the National Comprehensive Cancer Network guidelines, chemotherapy is recommended as part of the standard treatment for this case, in addition to post-mastectomy radiotherapy (National Comprehensive Cancer Network. NCCN Clinical Practice Guidelines in Oncology: Breast Cancer. Version 4.2025. Published April 2025. Accessed August 1, 2025. https://www.nccn.org/professionals/physician_gls/pdf/breast.pdf). For endocrine therapy, combination treatment with a CDK4/6 inhibitor should be considered. Alternatively, S-1 may be considered as a substitute for CDK4/6 inhibitors.^11)^ Based on these recommendations, we consulted with the patient, and considering her advanced age and personal preferences, she chose to receive endocrine therapy alone.

## CONCLUSIONS

In the present study, we describe a case of EPC that formed a cystic lymph node metastasis. Awareness of the possibility of such lymph node metastases in EPCs may assist with correct diagnosis and treatment in clinical practice.
